# Dosimetry Data Infrastructure for Individualized Radiopharmaceutical Therapy: International Experience and a Proposed Framework for China

**DOI:** 10.3390/ph19101641

**Published:** 2026-10-18

**Authors:** Qingqing Huang, Gang Huang

**Affiliations:** 1 Shanghai Key Laboratory of Molecular Imaging, Pudong Gongli Hospital, Shanghai University of Medicine and Health Sciences, Shanghai 201318, China; 2 Hangzhou Institute of Medicine, Chinese Academy of Sciences, Hangzhou 310018, China

**Keywords:** radiopharmaceutical therapy, individualized dosimetry, dosimetry database, quantitative imaging, real-world evidence, standardization, treatment decision support

## Abstract

**Background/Objectives:** There is increasing interest in moving selected radiopharmaceutical therapy (RPT) applications toward treatment informed by patient-specific quantitative data. Patient-specific dosimetry supports this transition, but routine implementation remains limited by methodological heterogeneity and incomplete linkage of absorbed dose with response, toxicity, and subsequent treatment decisions. This review examines evidence relevant to longitudinal dosimetry infrastructure and proposes a framework for China. **Methods:** This narrative review mapped evidence from established therapies, including ^177^Lu-DOTATATE, ^177^Lu-PSMA-617, ^131^I-NaI, and ^131^I-meta-iodobenzylguanidine (^131^I-MIBG), with ^90^Y microsphere selective internal radiation therapy used as a methodologically distinct comparator. International registries, quantitative imaging standards, and dosimetry resources were reviewed in parallel. **Results:** Available studies show substantial interpatient variation in absorbed dose and growing evidence of dose–response relationships. However, prescription, dosimetry methods, outcome definitions, and follow-up remain heterogeneous. Existing platforms address separate components of RPT care, but few link administration, pretherapy imaging, post-therapy quantitative imaging, dosimetry, clinical outcomes, toxicity, and later decisions across a complete treatment course. We propose seven core data domains and a three-tier architecture comprising hospital-based treatment decision platforms, regional dosimetry centers, and a national data center. **Conclusions:** A standardized longitudinal dosimetry infrastructure could improve multicenter comparability and provide a testable data foundation for individualized treatment, radiopharmaceutical development, and real-world evaluation. The proposed architecture is conceptual and requires staged consensus, pilot implementation, and prospective validation.

## 1. Introduction

Targeted radiopharmaceutical therapy (RPT) is increasingly used in oncology. ^177^Lu-DOTATATE is now an established treatment for neuroendocrine tumors, and the NETTER-1 and NETTER-2 trials have supported its use beyond later-line settings [1,2]. In prostate cancer, the TheraP and VISION trials supported the use of prostate-specific membrane antigen (PSMA)-targeted ^177^Lu-PSMA-617 in metastatic castration-resistant disease [3,4]. The European Association of Nuclear Medicine (EANM), the Society of Nuclear Medicine and Molecular Imaging (SNMMI), and professional organizations in China have also issued treatment guidelines, procedure standards, or expert consensus [5,6]. Together, these developments underscore the growing role of RPT in nuclear medicine practice.

RPT differs from external-beam radiotherapy in several important ways. After systemic administration, a radiopharmaceutical’s distribution is influenced by target expression, pharmacokinetics, tumor burden, organ function, product quality attributes, and patient-specific biology. Therefore, the same prescribed activity may yield different absorbed doses in tumors and normal organs. International Commission on Radiation Units and Measurements (ICRU) Report 96 identifies dosimetry-guided RPT as an important component of treatment optimization and emphasizes standardization in the prescription, recording, and reporting of dosimetric quantities [7]. EANM recommendations for ^177^Lu-labeled somatostatin receptor (SSTR)- and PSMA-targeted ligands also recognize the value of patient-specific dosimetry, while noting that clinical implementation remains limited by methodological complexity, available evidence, and local resources [8].

For this reason, the challenge is not only calculating dose but also applying dosimetry results in clinical care. These results need to be comparable across patients and institutions, interpretable over individual administrations and complete treatment courses, and linked to subsequent clinical decisions. In many centers, however, dosimetry is still performed primarily on an individual-case basis or within single-center workflows. Dose estimates are often stored separately from efficacy, toxicity, and treatment modification records, limiting the reuse of data generated during routine care [9,10].

In this review, “dosimetry database” refers to a data infrastructure spanning the entire RPT pathway, rather than a simple case registry or image archive. The intended contribution is not a new dose-calculation algorithm, but a longitudinal data architecture linking treatment eligibility, radiopharmaceutical administration, quantitative imaging, dosimetry, efficacy, safety, and subsequent clinical decisions. We define the evidence selection criteria, describe the current baseline of nuclear medicine in China, map representative clinical and dosimetric pathways, and examine gaps in existing international resources. These findings are then used to develop a conceptual national framework. The framework is proposed for further testing and validation and should not be interpreted as an established national standard.

## 2. Scope and Evidence Selection

This narrative review uses representative clinical and dosimetric evidence to inform a practical data architecture rather than to estimate pooled treatment effects. Therapies were selected based on three criteria: an established or clinically mature use with a defined treatment pathway; availability of patient-specific dosimetry or dose–effect evidence relevant to database design; and methodological features that illustrate different data requirements. The resulting evidence map covers ^177^Lu-DOTATATE, ^177^Lu-PSMA-617, ^131^I-NaI therapy for differentiated thyroid cancer (DTC), and ^131^I-meta-iodobenzylguanidine (^131^I-MIBG) therapy. ^90^Y microsphere selective internal radiation therapy (SIRT) was included as a methodologically distinct locoregional comparator because prospective randomized evidence supports the role of pretherapy dosimetric planning. However, its workflow is not directly interchangeable with that of systemic RPT. Emerging radionuclides, including ^161^Tb- and ^225^Ac-labeled agents, are considered only in relation to future data requirements and not as established standards of care.

Evidence was organized along the clinical pathway, beginning with oncologic diagnosis and prior treatment history and continuing through referral, confirmation of treatment indication, pretherapy target assessment, activity prescription, administration, post-therapy quantitative imaging and dosimetry, tumor and normal-organ absorbed doses, response, toxicity, follow-up, and subsequent treatment decisions. International registries, technical standards, dosimetry guidelines, and computational resources were examined in parallel because the proposed infrastructure must connect clinical and technical data within the same treatment pathway. Evidence was selected purposively, and the review was not designed as a systematic review or meta-analysis. The findings are therefore presented as an evidence map supporting a conceptual framework, with missing national or multicenter information identified explicitly as data gaps.

To improve transparency, targeted PubMed searches were conducted through 4 September 2026, with no prespecified lower publication date limit. Search concepts combined terms for radiopharmaceutical or radionuclide therapy with patient-specific dosimetry, absorbed dose, quantitative imaging, treatment planning, treatment response, toxicity, and the representative radiopharmaceuticals and radionuclides included in this review. Studies were purposively selected if they provided clinical, dosimetric, methodological, or data-infrastructure information relevant to the treatment pathways examined. The searches were designed to support a narrative evidence map rather than an exhaustive systematic review or quantitative synthesis.

## 3. Current RPT Practice and Data Infrastructure Needs in China

A national framework needs to reflect the scale and organization of current nuclear medicine practice in China. In 2024, the Chinese Society of Nuclear Medicine published a national survey covering calendar year 2023 and all 31 provincial-level regions in mainland China. The survey identified 1237 departments engaged in nuclear medicine-related work and recorded 772 positron-emission imaging systems, including 721 positron emission tomography/computed tomography (PET/CT) systems, and 1044 single-photon imaging systems, including 686 single-photon emission computed tomography/computed tomography (SPECT/CT) systems. The survey also reported 148 medical cyclotrons and a nuclear medicine workforce of 15,677. Radionuclide therapy was provided by 801 institutions, with 2993 dedicated treatment beds and 570,840 procedures under the survey’s broad definition of radionuclide therapy. Radioiodine (^131^I-NaI) treatment remained a major component: 718 institutions provided this treatment, including 103,780 procedures for Graves’ hyperthyroidism and 93,986 for DTC [11].

These data show that China already has a substantial clinical and imaging base for radionuclide medicine, but they also identify constraints relevant to a national dosimetry program. Sixty-six percent of surveyed departments reported workforce shortages, and 59% reported that regional economic conditions influence development [11]. These findings suggest that a tiered national model may be more practical than expecting every hospital to maintain the same imaging, medical physics, and dosimetry capabilities. At the same time, the national survey did not separately report contemporary ^177^Lu-based RPT volumes, the proportion of centers performing patient-specific post-therapy dosimetry, the availability of validated quantitative SPECT/CT workflows for therapy, RPT-specific medical-physics capacity, or longitudinal linkage of dosimetry with outcomes and treatment decisions. We also did not identify a publicly described nationwide registry that routinely captures these variables across radiopharmaceuticals. These missing data limit assessment of current dosimetry practice and support the need for a common national infrastructure.

The proposed framework is not intended to replace international dosimetry standards with a separate set of China-specific methods. Instead, it provides a national implementation structure through which established measurement and reporting principles could be applied across institutions with different levels of capability. Its main components include a common minimum dataset, traceable measurements, regional technical support, and interoperability with existing clinical and imaging systems. The features specific to China lie primarily in the implementation architecture, governance, and staged participation, while the underlying dosimetry principles remain aligned with international guidance.

## 4. Clinical–Dosimetric Evidence Map and Data Gaps

To place representative RPTs on a common basis, Table 1 summarizes the selected therapies using a common set of clinical and dosimetric dimensions: treatment eligibility, pretherapy imaging, activity prescription and administration, dosimetry, clinical outcomes, toxicity, follow-up, and subsequent treatment decisions. The following subsections examine the recurring data requirements and methodological gaps across these treatment pathways.

### 4.1. Prescription, Absorbed Dose, and Interpatient Variation

Fixed-activity administration is simple and can be implemented across institutions, but the same administered activity does not yield the same absorbed dose. Target expression, tumor volume, renal function, blood volume, body weight, clearance rate, and tumor burden can all affect radiopharmaceutical distribution and elimination, leading to marked variation in organ and lesion doses among patients. A systematic review and meta-analysis of ^177^Lu-PSMA dosimetry showed that this variation reflects not only patient biology but also imaging time points, reconstruction methods, segmentation, and the dosimetry workflow itself [25]. For treatment optimization, administered activity is the treatment input, whereas absorbed dose more directly reflects the radiation exposure received by tissue.

Clinical data show the extent of this variation. In an individualized ^177^Lu-DOTATATE study using repeated 7.4-GBq administrations at intervals of 8–12 weeks, the median renal absorbed dose was 0.61 Gy/GBq, with a range of 0.30–1.98 Gy/GBq, and the median absorbed dose per administration was 4.5 Gy, ranging from 2.2 to 14.3 Gy. Among patients who received treatment as planned, 73% were able to receive more than four administrations [12]. Other prospective studies further support the feasibility of dosimetry-guided treatment adaptation. In a 200-patient cohort, 7.4-GBq administrations were repeated until the renal absorbed dose reached 23 Gy or another stopping criterion was met; 123 patients reached 23 Gy after three to nine administrations [13]. In the P-PRRT trial, the first administered activity was individualized according to glomerular filtration rate and body surface area, with subsequent activities adjusted using the renal Gy/GBq derived from the preceding administration [15]. A subsequent phase II study similarly used renal dosimetry to individualize the number of 7.4-GBq administrations and prospectively evaluated response, survival, and toxicity [14]. These data illustrate why a multicenter database should retain both administered activity and cumulative organ dose rather than treating a nominal activity schedule as the treatment exposure itself.

### 4.2. Dose–Efficacy and Dose–Toxicity Evidence

For patient-specific dosimetry to inform clinical decisions, absorbed dose needs to be interpreted alongside efficacy and toxicity. In a prospective ^177^Lu-PSMA-617 cohort, the mean administered activity was approximately 7.8 GBq per treatment, and the median mean whole-body tumor absorbed dose was 11.55 Gy; patients achieving a ≥50% decline in prostate-specific antigen (PSA) had a median tumor dose of 14.1 Gy, compared with 9.6 Gy in nonresponders (*p* < 0.01). Only 1 of 11 patients with a tumor dose below 10 Gy achieved a ≥50% PSA decline [18]. A later lesion-level study in a larger cohort reported that a higher mean lesion dose was associated with better biochemical response and disease control, although the correlation was moderate and affected by biological heterogeneity among lesions [19]. In ^177^Lu-DOTATATE therapy, Warfvinge et al. described a dose–response relationship between cumulative tumor absorbed dose and tumor shrinkage [16]. Multicycle studies have also shown that tumor and organ pharmacokinetics may change between cycles, supporting assessment of dose–effect relationships over the full treatment course rather than at a single time point [17].

^90^Y microsphere SIRT provides a methodologically different example in which formal pretherapy planning is integral to the workflow. In DOSISPHERE-01, standard planning targeted 120 ± 20 Gy to the perfused lobe, whereas personalized planning targeted ≥205 Gy to the index lesion. The objective response rate at 3 months was 71% with personalized dosimetry versus 36% with standard dosimetry (*p* = 0.0074) [24]. Because SIRT is a locoregional treatment based on pretherapy vascular simulation, its workflow should not be treated as interchangeable with that of systemic RPT; nevertheless, it demonstrates why planned dose, administered activity, post-treatment distribution, delivered dose, and clinical outcome need to remain linked but distinct data elements.

In ^131^I-NaI therapy for DTC, blood/whole-body and lesion dosimetry, including approaches based on ^124^I PET/CT, have long been used in selected patients, while multicenter studies show substantial variation in organ absorbed dose and methodology [20,21,22].

^131^I-MIBG provides another established systemic model involving repeated treatment and patient-specific dosimetry. EANM guidance describes fixed activities of approximately 3.7–11.2 GBq, weight-based approaches up to 444 MBq/kg without stem-cell rescue and 666 MBq/kg when stem cells are stored, and protocols in which subsequent administrations are adapted according to whole-body absorbed dose. In a European survey cited in the guideline, 59% of centers providing ^131^I-MIBG therapy for neuroblastoma performed some dosimetry, but only 18% used dosimetry-based activity prescription [23].

Compared with the ^177^Lu-based therapies and ^90^Y SIRT discussed above, outcome-linked dosimetry data for ^131^I-NaI and ^131^I-MIBG are harder to compare across studies because treatment protocols, dosimetry methods, outcome definitions, and follow-up vary. The lack of harmonized dose–outcome data for these established therapies represents an important evidence gap and should not be interpreted as evidence that dosimetry lacks clinical value.

These results should not be interpreted as universal prescription thresholds. Rather, they show the information lost when administered activity, absorbed dose, response, toxicity, and treatment modification are stored separately. Lesion-level and multicycle studies also indicate that biological heterogeneity and changing pharmacokinetics can weaken simple dose–response relationships. A national database should therefore retain radionuclide- and drug-specific dose–effect data, toxicity definitions, and longitudinal treatment context before any clinical decision rule is developed.

### 4.3. Pretherapy Imaging, Dose Prediction, and Dosimetric Treatment Planning

Pretherapy imaging can serve three different purposes, which should be recorded separately in a database. It may confirm target expression and treatment eligibility, provide quantitative inputs for predicting therapeutic biodistribution or absorbed dose, or support formal dosimetric planning in which administered activity is selected to achieve a specified target or organ dose. These purposes are not interchangeable and require different levels of evidence. The broad label “theranostics” should not obscure the distinction between target verification, dose prediction, and treatment planning.

For PSMA-directed therapy, ^68^Ga-PSMA-11 PET can assess target expression before ^177^Lu-PSMA-617 treatment, although the diagnostic and therapeutic compounds differ in both radionuclide and ligand. Peters et al. found no significant correlation between lesion maximum standardized uptake value (SUV_max_) alone and subsequently measured lesion absorbed dose (*r* = 0.16, *p* = 0.47); even after incorporating kinetic assumptions, predicted-to-measured lesion dose ratios ranged from 0.4 to 2.7 [26]. Dose prediction also remains uncertain when the diagnostic and therapeutic agents share a closely related peptide backbone. Stenvall et al. reported only moderate tumor-level relationships between ^68^Ga-DOTATATE uptake and subsequent ^177^Lu-DOTATATE absorbed dose, with a 95% coverage interval from −65% to +248% for individual tumor-dose predictions [27]. Target expression on diagnostic imaging, therapeutic pharmacokinetics, and absorbed dose prediction should therefore be treated as related but distinct measurements.

The proposed data model should include a field specifying the purpose of pretherapy imaging: eligibility or target verification, prognostic characterization, quantitative dose prediction, or formal treatment planning. Other relevant fields include the diagnostic radionuclide and ligand, acquisition timing, quantitative imaging metric, prediction model, kinetic assumptions, uncertainty estimate, and validation status. If a predicted dose is used to guide treatment modification, the model version and decision rule should be recorded together with the final administered activity.

### 4.4. Clinical Outcomes, Follow-Up, and Treatment-Event Hierarchy

A database for dose–effect research requires more specific outcome definitions than a single “efficacy” field. For solid tumors, patient-level records should specify the response criteria used, the resulting category, and the date and imaging modality for each assessment. Categories may include complete response, partial response, stable disease, or progressive disease when assessment according to the Response Evaluation Criteria in Solid Tumors (RECIST) is appropriate. The database should also capture disease-specific biochemical markers such as PSA, duration of response, and the dates and clinical status needed to calculate progression-free and overall survival. Response and disease-control rates can then be calculated at the cohort level.

Toxicity records should specify the affected organ or system, the version and grade of the Common Terminology Criteria for Adverse Events (CTCAE), the dates of onset and recovery, relevant laboratory results, and whether the event altered subsequent treatment. Not all variables apply to every radiopharmaceutical or indication. The data dictionary should therefore distinguish among “not applicable,” “not assessed,” and “missing” rather than treating them as equivalent.

The treatment-event hierarchy also requires explicit identifiers. In this framework, an administration or session represents a single delivered radiopharmaceutical treatment event. A cycle or treatment block is a protocol-defined grouping of one or more administrations, whereas the treatment course comprises the full sequence of therapy for a given indication. When the literature uses “cycle” and “administration” interchangeably, the original terminology may be retained, provided that each administration is assigned a unique identifier.

Administered activity, imaging, and per-administration dosimetry should be linked at the administration level. Cumulative dose may be assigned to the relevant treatment block or the complete course, while toxicity and response assessments should be linked to their dates and corresponding treatment events. Reasons for delay, activity modification, or discontinuation should be recorded in a structured format. This hierarchy enables longitudinal data to reconstruct the clinical decision process rather than merely describe a sequence of outcomes.

### 4.5. Emerging Radionuclides and Future Data Requirements

A national data infrastructure should not be designed exclusively around ^177^Lu. The properties of ^161^Tb and ^225^Ac illustrate the additional data requirements introduced by emerging radionuclides. ^161^Tb emits beta particles as well as conversion and Auger electrons. Although quantitative ^161^Tb SPECT/CT is feasible, its additional short-range conversion and Auger electron emissions raise microdosimetric questions not fully captured by conventional organ-level absorbed-dose summaries [28]. ^225^Ac presents a different set of measurement and dosimetry challenges, including its multistep decay chain, daughter recoil and redistribution, low administered activities, and indirect quantification through gamma co-emissions [29].

Clinical and dosimetric evidence for these radionuclides remains less mature than for the established therapies discussed above. A national database should therefore accommodate radionuclide-specific metadata, assumptions about daughter-nuclide kinetics and redistribution, microdosimetric or radiobiological descriptors where relevant, and version-controlled calculation methods. This approach would allow the data model to evolve as new therapies and dosimetry methods emerge, rather than imposing a single schema derived from ^177^Lu therapy.

## 5. International Registries and Standardization Resources: Current Functions and Gaps

### 5.1. Clinical Data in Real-World Registries

The Radiopharmaceutical Therapy Registry (RaPTR), developed by SNMMI, is one of the publicly described projects that most closely resembles a dedicated RPT registry. RaPTR uses Research Electronic Data Capture (REDCap) to collect clinical information, including disease history, prior treatments, treatment response, and adverse events. RaPTR+PLUS extends the platform by using MIM Cloud to store de-identified PET, SPECT, and planar images. Publicly described modules include ^177^Lu-DOTATATE, ^177^Lu-PSMA-617, and ^131^I-NaI therapy for thyroid cancer [30].

This structure combines clinical and imaging data more extensively than a conventional disease registry. Access to raw or quantitative images is important if a registry is expected to support dosimetry rather than store only final dose estimates. Based on publicly available descriptions, however, dose calculation is not a primary function of RaPTR, and the clinical reasons for modifying treatment at a particular administration or treatment block are not presented as core structured variables.

The European Neuroendocrine Tumor Society (ENETS) Registry and the Spanish SEPTRALU study have addressed the issue from the perspective of disease registration and real-world outcomes. The ENETS Registry aggregates neuroendocrine tumor data from several European countries and has already supported long-term outcome analyses involving more than 10,000 patients [31]. SEPTRALU compiled multicenter real-world experience with ^177^Lu-DOTATATE in Spain and reported efficacy and safety across different primary tumor sites [32]. These systems are valuable because they capture clinical pathways and longer-term outcomes. However, dosimetry is usually not a central data layer, limiting direct analysis of dose–efficacy and dose–toxicity relationships. Quantitative imaging and absorbed-dose data may therefore be unavailable or recorded inconsistently, while the reasons for treatment modification may need to be reconstructed retrospectively rather than being entered prospectively as explicit decision variables.

### 5.2. Standardization Guidance and Dosimetry Resources

A substantial body of guidance and technical resources is already available for dosimetry standardization. Medical Internal Radiation Dose (MIRD) Pamphlet No. 23 established a general framework for patient-specific, three-dimensional quantitative SPECT [33], while MIRD Pamphlet No. 26 addressed radionuclide-specific requirements for quantitative ^177^Lu SPECT [34]. The EANM practice guideline for quantitative SPECT/CT emphasizes consistency in calibration, reconstruction, quality control, and clinical implementation [35]. The Radiation Dose Assessment Resource (RADAR) guides address internal dosimetry from complementary perspectives of data collection and data analysis [36,37]. EANM guidance on uncertainty analysis calls for identifying and propagating uncertainties through the dosimetry workflow [38]. ICRU Report 96, in turn, addresses standardization of dosimetry in prescription, recording, and reporting [7]. Both an earlier European survey and more recent reviews indicate that, despite growing methodological guidance, substantial variation remains in the extent to which dosimetry is implemented and in how workflows are organized across centers [39,40]. These documents define how quantitative imaging, dosimetry, uncertainty assessment, prescription, and reporting should be performed, but they are methodological standards rather than longitudinal clinical registries.

For dose calculation, OpenDose provides open access to standardized specific absorbed fractions, S values, and related dosimetric data [41]. MIRDcalc supports organ, suborgan, and tumor dosimetry within the MIRD framework and includes tools for calculation and quality checks [42]. These resources provide the methodological basis for quantitative imaging, dose calculation, and reporting. However, they do not follow patients longitudinally, capture efficacy and toxicity in routine practice, or record why a treatment was modified at a particular administration or treatment block. Their role is therefore complementary to that of a longitudinal clinical dosimetry infrastructure.

### 5.3. Gaps in Linking Dose, Outcomes, and Treatment Modification

The functions of existing resources are complementary. Clinical registries capture disease characteristics, treatment history, efficacy, and toxicity. Imaging extensions preserve source or quantitative imaging data. Methodological guidance defines how imaging and dosimetry should be performed, while dedicated software supports absorbed-dose calculation. A general platform that spans multiple radionuclides, diseases, and centers, and that also tracks dosimetry, outcomes, toxicity, and treatment decisions over time, remains uncommon.

The principal gap is not the absence of individual registries, standards, or calculation tools. It is the lack of a longitudinal structure linking treatment eligibility and pretherapy imaging with prescribed and administered activity, post-therapy quantitative imaging, absorbed dose, efficacy, toxicity, and the subsequent treatment decision for the same patient and treatment event.

This gap does not diminish the value of existing resources; it reflects the limits of their original functions. Registries are well-suited to real-world clinical data; quantitative imaging guidelines improve measurement consistency; dosimetry software provides algorithms and physical parameters; and clinical guidelines standardize treatment procedures. A national RPT dosimetry database in China would need to connect these functions so that clinical data, imaging, dosimetry results, outcomes, and decisions remain aligned at the level of the patient, administration or session, treatment block when applicable, and complete treatment course. The proposed framework is intended to provide this linkage rather than duplicate or replace an existing registry, guideline, imaging repository, or dosimetry software package.

## 6. Technical Prerequisites for a Multicenter Dosimetry Database

### 6.1. Traceable Activity Measurement and Radiopharmaceutical Administration Data

Net administered activity is an important input to dosimetry. The prescribed activity defines the treatment plan, whereas the activity actually delivered to the patient must be derived from measurements of the administration container before and after injection, with appropriate decay correction to the relevant times. The database should therefore capture the radionuclide, ligand, product batch, radiochemical purity, molar activity or activity per unit mass when relevant, calibration time, prescribed activity, measured pre-administration activity, residual activity after administration, extravasation, and net administered activity. Net administered activity should not be interpreted as a direct description of biodistribution. It defines the activity input to the dose calculation; subsequent distribution still depends on pharmacokinetics, target expression, tumor burden, and individual patient factors.

For multicenter use, traceability and calibration of activity measurements are essential requirements. If activity measurements across centers are not tied to a common calibration framework or a traceable standard, Gy/GBq values may not be comparable even when the same dosimetry equation is used.

### 6.2. Cross-System Consistency in Quantitative SPECT/CT

Quantitative SPECT/CT is a central component of post-therapy dosimetry for ^177^Lu. Systems from different manufacturers may differ in energy-window settings, scatter and attenuation correction, resolution recovery, iterative reconstruction, voxel size, and partial-volume correction. A multicenter study within the MRTDosimetry project showed that quantitative agreement across SPECT/CT systems can be substantially improved when clear calibration protocols and traceable activity measurements are used [43]. Therefore, a national database does not require every center to use the same scanner model. It does require each site to validate its quantitative workflow and demonstrate that its measurement error remains within an acceptable range.

### 6.3. Methodological Metadata Should Accompany Dose Results

A record stating only that “the kidney absorbed dose was 3.2 Gy” provides little insight into how that value was obtained. The database should also preserve the methodological metadata: imaging time points, volume-of-interest (VOI) segmentation method, lesion selection rules, organ mass, partial-volume correction, time-activity curve model, time integration method, S values, voxel kernels, Monte Carlo software version, and relevant uncertainty estimates. The International Atomic Energy Agency (IAEA) PLANETDose study showed appreciable inter-operator variation even when different operators analyzed the same patient data with the same software and a nominally standardized workflow [44]. For this reason, key intermediate results should remain available, and selected cases should undergo cross-review or secondary checks.

In resource-limited settings, the database should also support validated simplified dosimetry methods. Single-time-point SPECT/CT can provide useful dose estimates for selected radiopharmaceuticals and organs under defined conditions [45]. A 2026 study went further by optimizing single-time-point ^177^Lu-PSMA-617 dosimetry based on clinical outcomes rather than on physical accuracy alone [46]. Such simplified approaches should not be merged indiscriminately with multi-time-point reference methods. Their assumptions, permitted time windows, and validation ranges need to be documented so that later analyses can distinguish between them.

### 6.4. Standardized Dosimetry Reporting

A national database should store more than the final dose value. It should define a minimum dosimetry reporting dataset that includes prescribed and net administered activity; the treatment-course, block/cycle, and administration/session identifiers; imaging time points; quantitative calibration status; the source of organ and lesion VOIs; whether a dose is predicted/planned or measured post-therapy; absorbed and cumulative doses and their units; major uncertainties; the calculation algorithm and software version; whether a simplified method was used; and whether the result underwent review. For voxel-based dosimetry, additional variables may include dose-volume histograms (DVHs), D_90_/D_50_, maximum and mean doses, and relevant radiobiological indices. Recording the dose result with its method, temporal level, prediction status, and quality-control status makes the value more interpretable and comparable.

## 7. A Proposed National RPT Dosimetry Data Infrastructure for China

The framework presented below was developed from the recurring data requirements and gaps identified in Section 3, Section 4, Section 5 and Section 6. It remains conceptual and has not undergone formal stakeholder consensus, pilot implementation, or prospective clinical validation. The seven data domains and three-tier architecture should therefore be regarded as a starting model for testing and refinement rather than an established national standard.

### 7.1. Seven Core Data Domains

Despite differences among radiopharmaceuticals and treatment workflows, several data requirements recur. Treatment indication and prior therapy need to be linked to each administration. The purpose of pretherapy imaging should be distinguished from post-therapy imaging and dosimetry. Dose estimates require sufficient methodological metadata to support interpretation and comparison. Efficacy and toxicity need defined criteria and assessment times, while subsequent decisions must be linked to the relevant administration, treatment block, or complete course. These requirements are organized into seven core data domains in Table 2. These domains are proposed as a provisional core structure; their completeness, potential redundancy, and optimal granularity have not been formally established and will require stakeholder consensus and prospective evaluation.

### 7.2. A Three-Tier Architecture

The proposed infrastructure is organized into three functional tiers: a national data center, regional dosimetry centers, and hospital-based treatment decision platforms. These tiers describe functions rather than predefined administrative entities. Their governance, funding, and operational feasibility would need to be established through stakeholder consultation and pilot studies.

The national data center would primarily aggregate de-identified multicenter data, maintain a version-controlled common data dictionary, and establish national quality benchmarks. As the evidence base grows, it could also generate organ-dose reference ranges for Chinese populations, dose–efficacy and dose–toxicity models, and research datasets. Its purpose would not be to replace clinicians in selecting treatment. Rather, it would transform multicenter data into evidence resources that can be tested and reused.

Regional dosimetry centers would address uneven access to equipment, trained personnel, and medical physics support. Their work could include guidance on scanner and activity-meter calibration, validation of quantitative SPECT/CT protocols, VOI review, dosimetry for difficult cases, staff training, and feedback on data quality. Hospitals without a full medical physics team could use these centers to obtain more concentrated dosimetry support.

The hospital treatment decision platform would be positioned closest to clinical care. It could display a treatment overview, dosimetry reports, cumulative dose, trends in efficacy and toxicity, national or regional reference ranges, and a structured assessment for the subsequent administration or treatment block. The clinician would remain responsible for the treatment decision. The database would provide standardized information, longitudinal records, and risk signals rather than an automated prescription.

### 7.3. Tiered Data Submission: Start with a Minimum Viable Dataset

At the start of the program, it would be unrealistic to require every hospital to perform voxel dosimetry, biologically effective dose (BED) calculations, or complex uncertainty analysis. A tiered dataset is therefore more practical. Centers without established quantitative dosimetry workflows could submit a minimum dataset including baseline characteristics, radiopharmaceutical identity, net administered activity, available imaging information, hematologic and renal function measures, response assessments, toxicity, and subsequent treatment decisions. Dosimetry-specific fields could be recorded as unavailable or completed through support from a regional dosimetry center. Centers with validated quantitative imaging and dosimetry workflows could also additionally submit organ and lesion absorbed doses, while centers with more advanced equipment, staff, and computational support could contribute voxel-level dose, DVH, BED, lesion-level longitudinal tracking, and complete raw imaging data.

## 8. Potential Uses of the Database in Longitudinal Clinical Decision Making

### 8.1. Quality Control

Quality control is likely to be one of the first practical applications of the database. It requires less data and less model maturity than outcome prediction. Once local or multicenter reference distributions accumulate, an organ dose that falls well outside the expected range for comparable patients should prompt a review of net administered activity, imaging time points, SPECT/CT calibration, VOI definition, partial-volume correction, and time-activity curve fitting. At this stage, the database should primarily flag a potential data or workflow issue; it should not issue a treatment recommendation.

### 8.2. Developing Dose Reference Distributions for Chinese Patients

As the number of cases increases, dose distributions could be stratified by radiopharmaceutical, disease, organ function, tumor burden, age, body weight, and other relevant factors. This would enable the database to derive reference ranges for organ Gy/GBq, lesion dose, and cumulative dose. A reference built from clinically comparable patients is more informative than a single national average because it shows where a given patient falls within a group with similar clinical characteristics.

### 8.3. Recording the Reasons for Treatment Modification

Treatment-decision data should record what was changed, why it was changed, and the level of the treatment hierarchy at which the decision was made. For example, lower activity at a subsequent administration may reflect cumulative renal dose, thrombocytopenia, recent infection, patient preference, or disease progression. If the database records only “dose reduction,” clinically distinct situations become indistinguishable. A practical approach is to capture a structured primary reason, retain ranked contributing reasons and free text for nuance, and link the decision to the relevant session, treatment block, and overall treatment course.

### 8.4. Validation of Decision-Support Models

When the database is large enough to support predictive modeling, models for toxicity risk, treatment benefit, or subsequent-administration activity modification can be explored. Before such models are used in clinical prompts, they should undergo temporal validation, external validation across centers, and prospective silent validation, with ongoing monitoring for calibration drift. Multicenter data have shown substantial center-level differences in imaging protocols and renal absorbed-dose distributions [47]. Center, device, radiopharmaceutical, and workflow effects therefore need to be explicitly considered during model development and validation.

## 9. Data Governance, Interoperability, and Artificial Intelligence

### 9.1. Data Structure and Interoperability

The long-term value of a national database depends on whether its data can be reused across systems and research settings. The Findable, Accessible, Interoperable, and Reusable (FAIR) principles provide a useful framework for biomedical data management [48]. At the hospital level, demographics, laboratory results, imaging, and treatment records are typically stored in separate systems, including the hospital information system (HIS), laboratory information system (LIS), picture archiving and communication system/radiology information system (PACS/RIS), and radiopharmacy or radiopharmaceutical management system. Existing data should be retrieved through standard interfaces wherever possible to avoid duplicate data entry by clinicians. Structured clinical exchange can use Health Level Seven Fast Healthcare Interoperability Resources (HL7 FHIR) [49], whereas imaging remains anchored in Digital Imaging and Communications in Medicine (DICOM) and its quantitative metadata.

### 9.2. Privacy Protection and a Tiered Data Architecture

Nationwide clinical and imaging datasets are large and contain sensitive health information. The database would need to comply with Chinese laws and regulations governing personal information and data security [50], including de-identification, data minimization, role-based access, audit trails, and restrictions on secondary use. A tiered architecture may be appropriate for highly sensitive or high-volume data: metadata and key structured variables can be managed centrally, while raw images and full clinical records are accessed only when needed or analyzed in controlled environments. As infrastructure matures, federated analysis could also be considered, enabling multicenter models to be trained without copying every raw dataset into a single repository.

### 9.3. Appropriate Boundaries for Artificial Intelligence

In the early phase of database development, artificial intelligence (AI) is more naturally suited to workflow automation and quality control than to treatment prescription. Potential uses include automated organ and lesion segmentation, image registration across cycles, outlier detection, dosimetry workflow automation, and prompts for missing data. These tasks have relatively clear targets and can be checked by human reviewers. By contrast, a model that directly predicts “how much activity should be administered at the subsequent treatment event” carries substantially greater clinical risk and would require high-quality, standardized multicenter data with clear efficacy and toxicity labels. Until such evidence exists, AI output should be treated as a risk signal or as supporting information rather than as a substitute for clinical judgment.

## 10. A Staged Implementation Pathway for China

Implementation could begin with stakeholder consensus and development of the common data dictionary rather than immediate nationwide case collection. Nuclear medicine physicians, medical physicists, radiopharmacists, oncologists, imaging technologists, informatics specialists, methodologists, and data-governance representatives would need to agree on minimum fields, data definitions, acceptable measurement methods, outcome assessment times, and the treatment-event hierarchy. An initial module could focus on ^177^Lu-DOTATATE and ^177^Lu-PSMA-617. ^131^I-NaI, ^131^I-MIBG, and ^90^Y microsphere SIRT could be introduced as separate modules with therapy-specific fields rather than being fitted into an identical workflow.

The next stage would be a pilot involving a small group of centers with different levels of equipment, personnel, and dosimetry experience. Feasibility measures could include field completeness and patterns of missing data, the interval between care delivery and data submission, successful mapping from HIS, LIS, PACS, and radiopharmaceutical management systems, user workload, protocol adherence, and the proportion of cases for which the complete treatment course can be reconstructed. These measures would determine whether the proposed seven-domain model is practical before broader implementation.

Technical qualification could proceed alongside the pilot. Participating centers would be evaluated in activity measurement, quantitative imaging, and dosimetry reporting. Standard phantoms, traceable activity measurements, cross-system SPECT/CT validation, and common report templates could be used to assess intercenter reproducibility. Blinded cases and repeated analyses could help quantify variation related to operators and software. Regional dosimetry centers could support institutions that are initially unable to perform the complete workflow, allowing participation at different levels of technical capability.

After the pilot data model and technical qualification procedures have been refined, integration with hospital information systems could be expanded. Demographic data, laboratory results, imaging, radiopharmaceutical batch information, and administration records should be retrieved through standard interfaces where possible. Response assessment, attribution of toxicity, and the clinical reasons for treatment modification would still require clinician confirmation. Data-quality dashboards could return information on completeness, protocol deviations, calibration status, and unresolved outliers to participating centers.

Clinical-model validation would begin only after acceptable data comparability had been demonstrated. Models addressing dose–efficacy relationships, dose–toxicity relationships, simplified dosimetry, or treatment decisions could first be developed retrospectively and undergo temporal and external validation. This would be followed by prospective silent validation, in which model outputs are recorded without affecting patient care. Calibration, discrimination, center effects, and measures of clinical utility should be specified in advance. Only models that remain reproducible and adequately calibrated across independent centers should proceed to interventional decision-support studies.

This sequence separates validation of the data infrastructure from validation of clinical prediction models. Poor data completeness, incompatible fields, or unacceptable intercenter variation during the pilot would indicate that the data model or technical qualification process needs revision before wider implementation. Until prospective evidence supports a clinical decision role, the database should not function as an automated prescribing system. Its initial purpose is to provide standardized and traceable data through which clinical and dosimetric hypotheses can be tested.

## 11. Relevance to Radiopharmaceutical Development and Clinical Evaluation

### 11.1. Linking the Database to Radiopharmaceutical Development

The database’s value should extend beyond the clinical management of marketed RPT products. Radiopharmaceutical development depends on product quality attributes, target pharmacology, in vivo pharmacokinetics, and radiobiology. EANM guidance for first-in-human and early-phase studies already calls for recording key information, including activity measurements, pharmacokinetics, absorbed dose, and uncertainty [51]. If the national database is designed to accept early clinical study data, a new radiopharmaceutical could, in principle, be followed from first-in-human evaluation and dose escalation through registration studies and post-marketing real-world surveillance within a continuous data framework.

### 11.2. Improving Interpretation of Clinical Trial Results

Many RPT trials still rely on administered activity as the primary measure of treatment exposure, even though the same activity can yield markedly different tumor and organ doses. Including dosimetry as a companion variable can help distinguish inadequate drug effect from inadequate radiation delivery to target lesions and can clarify the source of normal-organ toxicity. A longitudinal infrastructure could also preserve the distinction between eligibility imaging, predicted/planned dose, and measured post-therapy dose. As the database grows, quality-controlled multicenter dose distributions could inform dose exploration and the design of future clinical studies.

### 11.3. Evaluation of Product Quality, Imaging Systems, and Software

Clinical dosimetry for the same radiopharmaceutical is shaped not only by the drug but also by the imaging system, reconstruction workflow, and dosimetry software. The database could be used to detect systematic shifts in dose distributions following a product batch change, scanner upgrade, or algorithm update. Evaluation of domestically developed SPECT/CT systems, dosimetry software, and automated segmentation tools could therefore extend beyond phantom testing to include quality-controlled multicenter real-world data.

### 11.4. Data for Reimbursement and Health Technology Assessment

Individualized dosimetry adds imaging, medical physics, and data-processing workloads. For it to become a routinely reimbursed service, evidence will be needed that it reduces serious toxicity, avoids ineffective treatment cycles, improves attainment of the target dose, or uses health-care resources more efficiently. A national database could provide real-world evidence to address these questions, but reimbursement decisions should still rest on validated clinical benefit rather than on the completion of a dosimetry calculation alone.

## 12. Discussion

The current scale of nuclear medicine practice in China provides a basis for testing a national RPT dosimetry infrastructure. The 2024 national survey documented a large clinical network, including 801 institutions providing radionuclide therapy and substantial SPECT/CT capacity, while also showing workforce shortages and regional economic constraints [11]. However, the survey did not report national use of patient-specific dosimetry or the extent to which dosimetry data are longitudinally linked to outcomes and treatment decisions. The proposed framework addresses this information gap by defining the data that need to be connected and the treatment-event level at which they should be recorded. Increasing case numbers alone would not produce comparable evidence without common definitions, measurement standards, and longitudinal linkage.

Given the uneven distribution of equipment, personnel, and medical physics support, implementation should combine common standards with tiered participation. Centers with limited resources could begin by recording variables that are relatively straightforward to standardize, including net administered activity, defined post-therapy imaging, laboratory results, clinical outcomes, and treatment decisions. Regional dosimetry centers could provide support for quantitative imaging and dose calculation, while centers with greater technical capacity could undertake voxel-level dosimetry and methodological validation. ICRU Report 96 similarly uses graded approaches to dosimetry and reporting for different resource settings [7]. A model that allows institutional capability to develop over time may be more practical in China than a single high entry threshold.

The proposed framework has several limitations. Evidence was selected purposively for a narrative evidence map rather than through a systematic review, and relevant studies may therefore have been omitted. The framework was not developed through a formal Delphi process or guideline-development procedure, has not undergone prospective pilot testing, and was not evaluated using a new patient dataset. The national baseline also remains incomplete. The Chinese survey provides nationwide data on departments, equipment, personnel, and broadly defined radionuclide therapy volumes for 2023, but it does not report current national treatment volumes for individual ^177^Lu therapies or the adoption of patient-specific dosimetry [11]. Evidence for dose–efficacy and dose–toxicity relationships also differs among radiopharmaceuticals, indications, organs, and dosimetry methods. A threshold derived for one drug or organ should not be transferred directly to another treatment setting. The initial role of the database should therefore be to generate and test evidence rather than encode unvalidated clinical decision rules. Importantly, establishing a longitudinal dosimetry database would enable evaluation of dose–outcome relationships and dosimetry-guided treatment strategies, but it would not, in itself, demonstrate improved clinical outcomes. The clinical benefit of dosimetry-guided decision-making would need to be assessed separately in prospective studies.

International resources already provide important components of the required infrastructure. RaPTR, ENETS, and SEPTRALU contribute registry and real-world clinical data; EANM, MIRD, and ICRU documents provide methodological and reporting guidance; and OpenDose and MIRDcalc support dose calculation. The proposed Chinese framework is intended to complement, not replace, these resources. Its distinctive feature is the longitudinal linkage of eligibility imaging, dose prediction or treatment planning, administered treatment, post-therapy dosimetry, standardized outcomes, toxicity, and subsequent decisions within a defined treatment-event hierarchy. Whether this architecture improves data completeness, multicenter comparability, or the development of clinically useful models remains to be determined through consensus building, pilot implementation, and prospective validation.

## 13. Conclusions

There is increasing interest in using patient-specific quantitative data to inform selected RPT applications. Evidence increasingly links absorbed dose with response in several therapies, but clinical translation remains limited by heterogeneous imaging and dosimetry methods, fragmented records, uncertainty in predicting therapeutic absorbed dose from diagnostic imaging, and incomplete documentation of decisions across treatment administrations. A national dosimetry infrastructure in China should link treatment eligibility and radiopharmaceutical administration with quantitative imaging, planned, predicted, or measured dose, standardized efficacy and toxicity assessments, follow-up, and subsequent clinical decisions. Each data element should be linked to the relevant administration or session, treatment block when applicable, and complete treatment course.

This review outlines seven core data domains, a three-tier architecture, tiered data submission, and a staged validation pathway. Because the framework remains conceptual, the immediate priorities are stakeholder consensus on definitions, technical qualification, pilot implementation, quality control, and demonstration of multicenter comparability. Population-specific dose reference distributions, dose–efficacy and dose–toxicity models, and prospective decision-support tools should be developed only after these requirements have been met. The resulting longitudinal data could also support radiopharmaceutical development, clinical evaluation, device and software validation, and real-world health technology assessment.

## Figures and Tables

**Table 1:** Standardized clinical–dosimetric evidence map for representative radiopharmaceutical therapies.

Therapy/ Indication	Eligibility and Pretherapy Imaging	Prescription/ Administered Activity	Dosimetry, Outcomes, and Toxicity (Representative Evidence)	Follow-Up/Implications for Subsequent Treatment and Database Design
^177^Lu-DOTATATE/SSTR-positive neuroendocrine tumors	SSTR imaging and clinical criteria establish treatment eligibility. Pretherapy imaging is used primarily to confirm target expression.	Fixed-activity regimens are established. Dosimetry-guided studies have used 7.4 GBq every 8–12 weeks, with the number of administrations adjusted according to renal absorbed-dose or BED limits and other stopping criteria [12,13,14]. In the P-PRRT trial, the first administered activity was individualized according to GFR and body surface area, with subsequent administrations adjusted according to the prior renal Gy/GBq [15].	In one individualized study, the median renal absorbed dose was 0.61 Gy/GBq (range, 0.30–1.98 Gy/GBq), and the median dose per administration was 4.5 Gy (range, 2.2–14.3 Gy). Among patients who received treatment as planned, 73% received more than four administrations. Tumor dose–response relationships have also been reported [12,16,17].	Linkage of each administration with renal and tumor doses, hematologic and renal toxicity, imaging response, cumulative dose, and the reason for continuing, delaying, modifying, or discontinuing treatment.
^177^Lu-PSMA-617/metastatic castration-resistant prostate cancer	PSMA PET confirms target-positive disease, but pretherapy PET measurements are not automatically quantitative surrogates for therapeutic absorbed dose.	Clinical trials and routine protocols generally use a fixed activity per administration. A prospective dosimetry cohort used a mean administered activity of approximately 7.8 GBq [18].	The median whole-body tumor absorbed dose was 11.55 Gy. Patients with a ≥50% PSA decline had a median tumor dose of 14.1 Gy, compared with 9.6 Gy among nonresponders (*p* < 0.01). Lesion-level dose–response relationships were affected by biological heterogeneity [18,19].	Separate recording of baseline PSMA PET and post-therapy quantitative imaging and dosimetry, with lesion and cumulative doses linked to PSA changes, imaging response, organ toxicity, and subsequent treatment decisions.
^131^I-NaI/differentiated thyroid cancer	Clinical risk and radioiodine avidity determine eligibility. ^123^I or ^124^I imaging may be used in selected diagnostic, dose-prediction, or treatment-planning workflows.	Empirical activity prescription and patient-specific blood, whole-body, or lesion dosimetry are used in selected settings [20,21].	Blood or marrow, whole-body, lesion, and normal-organ dose estimates depend on the methods used. Multicenter studies have shown substantial interinstitutional variation in organ absorbed doses [22]. Comparable dose–response and dose–toxicity relationships remain difficult to define because dosimetry methods, clinical outcome definitions, and follow-up are heterogeneous.	Longitudinal linkage of treatment intent, administered activity, clearance kinetics, blood or marrow dose, whole-body and lesion doses, biochemical and imaging response, toxicity, and the reason for repeated treatment.
^131^I-MIBG/neuroblastoma and selected MIBG-avid neuroendocrine tumors	MIBG-avid disease is required. Pretherapy assessment supports eligibility and, in selected protocols, treatment planning.	Approaches include fixed activities of approximately 3.7–11.2 GBq, weight-based activities of up to 444 MBq/kg without stem-cell rescue and 666 MBq/kg when stem cells are stored, and protocols based on whole-body absorbed dose [23].	Whole-body absorbed dose may be used as a surrogate for marrow exposure. In a European survey, 59% of centers performed some form of dosimetry, but only 18% used dosimetry to prescribe administered activity [23]. Comparable dose–response and dose–toxicity estimates remain difficult to derive across heterogeneous treatment protocols and outcome definitions.	Linkage of repeated administrations with whole-body and tumor doses, marrow and liver toxicity, pharmacokinetics, and the effect of measurements from the first administration on subsequent prescribed activity.
^90^Y microsphere SIRT/unresectable hepatocellular carcinoma	Angiographic assessment and ^99m^Tc-MAA simulation are used to evaluate shunting and support treatment planning.	In DOSISPHERE-01, standard planning targeted 120 ± 20 Gy to the perfused lobe, whereas personalized planning targeted ≥205 Gy to the index lesion [24].	The objective response rate at 3 months was 71% with personalized dosimetry and 36% with standard dosimetry (*p* = 0.0074). Its workflow and dose metrics differ from those of systemic RPT [24].	Separate but linked recording of simulation findings, planned target and normal-liver doses, administered activity, post-therapy activity distribution, delivered dose, response, toxicity, and discrepancies between planned and delivered treatment.

Note: RPT, radiopharmaceutical therapy; SSTR, somatostatin receptor; PSMA, prostate-specific membrane antigen; PSA, prostate-specific antigen; SIRT, selective internal radiation therapy; BED, biologically effective dose; MIBG, meta-iodobenzylguanidine; MAA, macroaggregated albumin; GFR, glomerular filtration rate; P-PRRT, personalized peptide receptor radionuclide therapy.

**Table 2:** Proposed core data domains and representative fields.

Data Domain	Minimum Fields (Examples)	Extended Fields (Examples)	Primary Use
1. Patient baseline	Disease type, treatment indication, stage or grade, performance status, organ function, blood counts, prior therapy, treatment-course identifier, target-expression imaging and eligibility status, tumor burden	Genomic or molecular data, comorbidities, marrow risk, organ volumes	Eligibility assessment, risk stratification, and comparison with clinically similar patients
2. Radiopharmaceutical and administration	Radionuclide, ligand, product batch, radiochemical purity, prescribed activity, administration time, net administered activity, administration route, unique session identifier, and parent cycle or treatment-block identifier when applicable	Molar activity or activity per unit mass, additional product quality attributes, quantitative assessment of extravasation	Documentation of treatment input, treatment-event hierarchy, and product quality
3. Quantitative imaging	Purpose of pretherapy imaging, diagnostic radionuclide and ligand, post-therapy imaging, acquisition times, scanner, reconstruction method, and calibration factor	Raw DICOM data, correction methods, recovery coefficients, prediction model and assumptions, model version, and quantitative quality-control results	Target verification, pharmacokinetic analysis, and cross-center comparison without conflating diagnostic imaging with dose prediction
4. Dosimetry	Status as planned, predicted, or measured dose; organ-at-risk dose; representative lesion dose; cumulative dose; calculation method; and uncertainty estimate	Voxel-level dose, dose-volume histogram, biologically effective dose, relevant microdosimetric or radiobiological descriptors, and daughter-nuclide assumptions	Dose–response analysis, method comparison, and treatment adaptation
5. Efficacy and toxicity	Response criteria and category, disease-specific biomarkers, follow-up date and method, blood counts, liver and renal function, CTCAE version and grade, and toxicity onset and recovery	Progression date and status, survival status, date of death or last follow-up, response duration, patient-reported outcomes, and long-term organ function	Analysis of dose–efficacy and dose–toxicity relationships using defined endpoints
6. Treatment decision	Continue treatment, delay treatment, modify activity, change therapy, or discontinue treatment; decision level at session, block, or course; and primary reason	Multidisciplinary discussion, ranked contributing factors, patient preference, and alternative treatment	Reconstruction of why treatment changed over the complete course
7. Quality control	Data completeness, compliance with imaging time points, quantitative validation, review status, and outlier handling	Center capability level, annual phantom or blinded-case results, and intercenter reproducibility measures	Improvement of multicenter data quality and qualification of centers for more advanced analyses

Note: DICOM, Digital Imaging and Communications in Medicine; CTCAE, Common Terminology Criteria for Adverse Events.

## Data Availability

No new data were created or analyzed in this study. Data sharing is not applicable to this article.
